# Mechanisms of Resistance to Trastuzumab and Novel Therapeutic Strategies in HER2-Positive Breast Cancer

**DOI:** 10.1155/2012/415170

**Published:** 2012-05-09

**Authors:** Andrea L. A. Wong, Soo-Chin Lee

**Affiliations:** ^1^Department of Haematology-Oncology, National University Cancer Institute, National University Health System, Singapore 119228; ^2^Cancer Science Institute, National University of Singapore, Singapore 117599

## Abstract

HER2-positive breast cancers have poorer prognosis and are prime candidates for molecular-targeted therapy because they are driven by the unique mechanism of HER2 oncogene addiction. While anti-HER2 agents such as trastuzumab and lapatinib are integral to the treatment of HER2-positive breast cancer, intrinsic and secondary resistance pose a significant challenge, underscoring the need to develop novel anti-HER2 therapies. In recent years, an array of promising and novel anti-HER2 therapeutic agents and their combinations have entered various stages of clinical development. However, questions remain on the optimal sequences of HER2-directed therapies and selection of patients for the most appropriate drug or combinations; incompletely defined mechanisms of trastuzumab action and resistance have also dampened the progress of more successful biomarker-driven treatment approaches. This paper summarizes existing preclinical and clinical evidence on the mechanisms of trastuzumab action and resistance and provides an up-to-date overview of novel HER2-directed therapies in clinical development.

## 1. Background

Human-epidermal-growth-factor-receptor-2 (HER2-) overexpressing breast cancers account for 20–25% of invasive breast cancers and are associated with an aggressive biologic behaviour translating to poorer clinical outcomes [1]. The development of trastuzumab, a recombinant humanised monoclonal antibody targeting the extracellular domain (ECD) of the HER2 protein, has dramatically altered the natural history of HER2-positive breast cancer and ranks among the most significant advances in breast cancer therapeutics. Trastuzumab was originally approved for use in HER2-positive metastatic breast cancer by the United States Food and Drug Administration (FDA) in 1998, based on a randomised phase III study, where the combination of trastuzumab and chemotherapy in previously untreated patients significantly improved objective response rates (ORR), progression-free survival (PFS), and overall survival (OS) over chemotherapy alone [2]. FDA approval for the use of trastuzumab in the adjuvant setting was obtained in 2006, based on an interim analysis of two National Cancer Institute-Cooperative Group trials (NSABP 31 and NCCTG N9831) demonstrating remarkable prolongation of disease-free survival (DFS) with the addition of trastuzumab to chemotherapy in HER2-positive early breast cancer [3].

 Despite this notable success, 70% of patients with HER2-positive breast cancers demonstrate intrinsic or secondary resistance to trastuzumab [4], highlighting the importance of developing new therapies for this disease. This paper aims to explore the possible mechanisms of trastuzumab resistance, provide an overview of the myriad of HER2-directed therapeutic options that have entered active clinical development in recent years, and examine their implications on the future management of HER2-positive breast cancer.

 The data for this paper were obtained by searching the PubMed database using Entrez. The search terms used included the following combined subject headings: HER2-positive breast cancer, herceptin, trastuzumab, resistance, p95HER2, phosphatidylinositol 3-kinase (PI3K)/Akt, phosphatase and tensin homolog (PTEN), HER3, insulin-like growth factor 1 receptor (IGF-1R), angiogenesis, lapatinib, pertuzumab, trastuzumab-DM1 (T-MD1), HER2 tyrosine kinase inhibitors (TKIs), heat-shock protein (HSP) 90 inhibitors, vascular endothelial growth factor (VEGF) inhibitors, IGF-1R inhibitors, and bispecific antibodies. The citation lists of all retrieved articles were examined to identify potentially relevant articles, and proceedings from conferences of the American Society of Clinical Oncology and San Antonio Breast Cancer Symposium were searched for relevant abstracts.

## 2. Mechanisms of Action and Resistance to Trastuzumab

### 2.1. Epidermal Growth Factor Receptor Family Signalling Pathway

HER2 belongs to a family of transmembrane receptor tyrosine kinases, which also includes HER1 (also known as epidermal growth factor receptor (EGFR)), HER3, and HER4. Ligand binding to the ECD of these receptors results in either homodimerisation between two molecules of the same receptor or heterodimerizaton between two different receptors. Dimerisation in turn induces tyrosine kinase phosphorylation and downstream signalling onto the PI3K and mitogen-activated protein (MAP) kinase cascades, leading to cell survival and proliferation, respectively. While EGFR, HER2, and HER3 are all implicated in carcinogenesis, the dimers vary in their signalling potencies, with the HER2/HER3 heterodimer possessing the strongest mitogenic potency, particularly in the activation of the PI3K/Akt survival pathway. The HER2 protein possesses two unique features; firstly, unlike other HER family members which exist in an inactivated state, it can be constitutively activated and is capable of ligand-independent dimerisation, and secondly, it is the preferred heterodimerisation partner for other HER proteins [5, 6]. 

### 2.2. Mechanisms of Action of Trastuzumab

Ironically, the mode of action of trastuzumab remains incompletely defined despite its routinary clinical application. Extensive preclinical research has been conducted to elucidate these mechanisms, and the following possibilities have been proposed.

#### 2.2.1. Immune-Mediated Response

An important proposed mechanism of action of trastuzumab is antibody-dependent cellular cytotoxicity (ADCC), which is triggered through the detection of Fc portion of trastuzumab by the Fc*γ* receptor on immune effector cells, particularly natural-killer cells, resulting in cell lysis of HER2-positive target cells bound to trastuzumab [5]. This mechanism of action is supported by findings from a preclinical study of HER2-overexpressing murine xenograft models, in which the antitumour activity of trastuzumab was markedly reduced in mice bearing defective Fc*γ* receptors [7]. These observations are further reinforced by *in vivo* data from a pilot study of 11 patients with HER2-positive early breast cancer, where a positive correlation was observed between response to neoadjuvant trastuzumab and ADCC activity [8].

#### 2.2.2. Inhibition of HER2 Proteolytic Cleavage

p95HER2 fragments resulting from the proteolytic cleavage of the HER2 ECD have generated much interest due to the association of the 100–115 kDa p95HER2 fragment with a clinically more aggressive subset of HER2-positive breast cancers [4]. A preclinical study has demonstrated that trastuzumab inhibits HER2 ECD cleavage through the proposed mechanism of steric hindrance [9], and several clinical studies have shown correlation between trastuzumab-induced decline in serum HER2 ECD levels with improved tumour response and PFS, lending support to this hypothesis [10, 11].

#### 2.2.3. Inhibition of Signal Transduction Pathways and Cell-Cycle Progression

The role of trastuzumab in abrogating the PI3K/Akt signaling pathway has been addressed in two preclinical studies, where treatment of HER2-gene-amplified breast cancer cells with trastuzumab caused growth inhibition through PTEN upregulation and downregulation of PI3K activity and Akt function [12, 13]. Other studies have suggested that trastuzumab also exerts its influence further downstream the cell signalling cascade, causing G1 phase cell-cycle arrest through upregulation of cyclin-dependent kinase (cdk) inhibitor p27^Kip1^ expression [14, 15].

#### 2.2.4. Inhibition of Angiogenesis

Investigators have reported that HER2-overexpressing cell lines possess higher basal levels of VEGF expression and that stimulation with the HER3 and HER4 ligand, heregulin *β*1, further enhances VEGF secretion [16], suggesting that upregulation of angiogenesis may contribute to the tumorigenicity of HER2-positive breast cancer. Trastuzumab induced normalisation and regression of the vasculature in HER2-overexpressing breast cancer xenografts [17], whilst in a separate study, the combined administration of trastuzumab and paclitaxel resulted in the best response in tumour models compared to either treatment alone, indicating that trastuzumab-induced normalisation of tumour vasculature may have permitted more efficient intratumoural drug delivery, resulting in synergistic activity of the combination [18].

### 2.3. Mechanisms of Trastuzumab Resistance

Understanding the mechanisms behind trastuzumab resistance is a critical step towards the development of novel anti-HER2 strategies. Molecular mechanisms that contribute to trastuzumab resistance include the following.

#### 2.3.1. Truncated HER2 Receptor

Truncated p95HER2 fragments exhibit resistance to trastuzumab because they lack trastuzumab-binding epitopes and may arise either through proteolytic cleavage of the HER2 ECD or alternative translation-initiation sites of the HER2 protein [4]. Due to their propensity to cause more rapid and acute activation of mitogenic signaling cascades, expression of 100–115 kDa p95HER2 fragments in the mammary glands of transgenic mice resulted in far more aggressive and invasive breast tumours compared to those driven by the full-length receptor [19]. As such, p95HER2 has a potential role both as a prognostic and predictive biomarker. Indeed, patients with breast cancers overexpressing p95HER2 were found to have a higher incidence of lung metastases and experienced significantly shorter PFS and OS with trastuzumab treatment, compared to patients expressing only the full-length receptor [20].

#### 2.3.2. Aberrant Activation of Downstream Signalling Pathways

Genetic aberrations in the PI3K/Akt pathway are among the most prevalent in breast cancer and have been shown to mediate trastuzumab resistance. These include loss-of-function PTEN deletions and activating mutations of PI3KCA, the gene encoding the p110 catalytic subunit of PI3K [21]. A preclinical study employing a large-scale functional RNA interference screen detected PTEN as the main modulator of trastuzumab sensitivity in a HER2-overexpressing breast cancer cell line. Furthermore, both PTEN knockdown as well as transfection of the cell line with a PI3KCA mutation rendered it insensitive to trastuzumab [22]. In concordance, both PIK3CA mutations and PTEN loss were associated with inferior time-to-progression and survival in a retrospective study of 256 trastuzumab-treated HER2-positive metastatic breast cancer patients [23].

#### 2.3.3. Compensatory Activation of Parallel Signalling Pathways

Inhibition of the HER2 oncogenic pathway with trastuzumab may result in compensatory crosstalk and activation of alternative signalling pathways, such as IGF1R and HER3 signalling pathways [24]. Aberrant activation of IGF1R signalling was the first mechanism of trastuzumab resistance to be described. In a preclinical study, co-expression of HER2 and IGF1R in breast cancer cells resulted in loss of sensitivity to trastuzumab treatment, whereas blocking ligand activation of IGF1R restored trastuzumab-related growth inhibition [25]. In addition, upregulation of HER3 signalling and consequent activation of the PI3K/Akt pathway has been demonstrated upon exposure of HER2-positive breast cancer cell lines to HER TKIs [26]. In concordance, the HER2/HER3 dimerisation inhibitor, pertuzumab, exhibited activity in breast cancer cells resistant to trastuzumab through potent inhibition of HER3 ligand-induced morphogenesis, lending support to the hypothesis that compensatory activation of this pathway is a key mediator of trastuzumab resistance [6, 27].

#### 2.3.4. Steric Hindrance of Receptor-Antibody Interaction

The binding between trastuzumab and HER2 may be disrupted by the membrane-associated glycoprotein mucin-4 (MUC4), as evidenced by the overexpression of MUC4 in a trastuzumab-resistant breast cancer cell line and subsequent restoration of trastuzumab binding through MUC4 siRNA knockdown [28]. Similarly, another preclinical study demonstrated that a breast cancer cell line acquired trastuzumab resistance through the upregulation of a cleaved form of the MUC1 protein, MUC1*, and resistance was reversed by MUC1* antagonists [29].

## 3. Treatment of Trastuzumab-Resistant HER2-Positive Breast Cancer

Only two agents possess regulatory approval for the treatment of HER2-positive breast cancer, with a lull of nearly a decade between the initial FDA approval of trastuzumab and that of lapatinib in 2007. Therefore, the management of patients with prior trastuzumab failure has long represented an area of unmet clinical need, with existing treatment options that include HER2-directed therapies being limited to (i) the continuation of trastuzumab beyond progression, (ii) the alternative use of lapatinib, or (iii) a combination of both.

### 3.1. Existing Strategies in the Management of Trastuzumab-Resistant Disease

#### 3.1.1. Trastuzumab Beyond Progression

Preclinical data showing that trastuzumab withdrawal resulted in rapid re-growth of trastuzumab-resistant cell lines [30, 31] lent support to the strategy of continuing trastuzumab beyond disease progression in clinical practice. Although frequently utilised by breast cancer physicians, the evidence for this was previously substantiated only by retrospective clinical data [32, 33]. Only recently did results from a prospective phase III study emerge, demonstrating that continuing trastuzumab in combination with capecitabine in HER2-positive metastatic breast cancer patients who had progressed on trastuzumab was superior to treating with capecitabine alone, with improved ORR (48.1% *versus* 27%, *P* = 0.0115) and median time-to-progression (8.2 months *versus* 5.6 months, *P* = 0.0338) [34]. Data from other prospective studies examining the same issue but using different chemotherapeutic regimens are awaited (Pandora, *NCT00444587*; THOR, *NCT00448279*) [35].

#### 3.1.2. Lapatinib

Lapatinib is a reversible small-molecule TKI of EGFR and HER2 which was first found to possess antitumour activity in HER2-dependent cells lines [36]. Subsequent preclinical and retrospective clinical studies have also indicated its potential activity in the subset of p95HER2-overexpressing, trastuzumab-resistant breast tumours [37, 38].

Lapatinib gained regulatory approval for use in HER2-positive metastatic breast cancer patients who have received prior anthracyclines, taxanes, and trastuzumab, based on a pivotal phase III study demonstrating the superiority of lapatinib and capecitabine compared to capecitabine alone in ORR (22% *versus* 14%, *P* < 0.09) and PFS (8.4 months *versus* 4.4 months, *P* < 0.001) in this group of patients [39]. In addition, lapatinib has demonstrated promising activity in central nervous system (CNS) metastases, which affect one-third of patients with advanced HER2-positive breast cancer. The high incidence of CNS metastases in HER2-positive breast cancer is in part contributed by the inability of trastuzumab to penetrate the blood-brain barrier. In a multi-centre phase II study, 20% of patients with refractory brain metastases achieved a CNS objective response following treatment with lapatinib and capecitabine [40], highlighting the promise of a small molecule TKI in this subset of patients.

 Lapatinib is currently being evaluated in combination with chemotherapy as first-line metastatic treatment in HER2-positive disease in the ongoing MA31 study, where it is being compared to trastuzumab in combination with taxane-based chemotherapy (*NCT00667251*). In addition, a randomised phase II study has been planned to evaluate the efficacy of lapatinib versus trastuzumab in combination with first-line chemotherapy in metastatic patients whose tumours display concomitant overexpression of HER2 and p95HER2 (*NCT01137994*). Lapatinib is also being evaluated as monotherapy or in combination with trastuzumab in early-stage breast cancer in the adjuvant (ALTTO, *NCT00490139; *TEACH, *NCT00374322*) and neoadjuvant (neoALTTO, *NCT00553358; *GeparQuinto, *NCT00567554*) settings.

#### 3.1.3. Lapatinib and Trastuzumab

A preclinical study in HER2-overexpressing breast cancer cell lines suggested that dual anti-HER2 blockade was synergistic because lapatinib induced HER2 accumulation at the cell surface, resulting in enhanced trastuzumab-binding and increased trastuzumab-mediated ADCC [41]. A randomised phase III study conducted in heavily pretreated HER2-positive metastatic breast cancer patients who progressed on trastuzumab demonstrated that the combination of trastuzumab and lapatinib significantly improved clinical benefit rates (CBR) (24.7% *versus* 12.4%, *P* = 0.01) and median PFS (12 weeks *versus* 8.1 weeks, *P* = 0.008) compared to lapatinib alone [42]. These findings confirmed the efficacy of dual anti-HER2 blockade in the context of a chemotherapy-free regimen associated with minimal toxicities. Recently, preliminary data from the neoALTTO study suggested that dual anti-HER2 blockade combined with paclitaxel chemotherapy can further improve treatment efficacy, resulting in significantly higher pathologic complete response (pCR) rates than paclitaxel combined with either trastuzumab or lapatinib alone in the neoadjuvant setting (51.3% *versus* 29.5% *versus* 24.7%, respectively; *P* < 0.01); dual blockade was associated with mildly increased but manageable toxicities [43].

### 3.2. Novel HER2-Directed Therapies (Table 1)

The emergence of more robust preclinical data in recent years has catapulted therapeutic advances in this arena, resulting in a rapid expansion in the armamentarium of anti-HER2 agents being developed clinically. These include dimerisation inhibitors, antibody-drug conjugates, tyrosine kinase inhibitors, HSP90 inhibitors, mTOR/PI3K inhibitors, antiangiogenic agents, IGF-1R inhibitors, and bispecific antibodies.

#### 3.2.1. Dimerisation Inhibitor, Pertuzumab

One of the closest to regulatory approval amongst several novel anti-HER2 agents in advanced clinical development is pertuzumab, which is a humanised monoclonal antibody that binds to an epitope on the dimerisation domain located on domain II of the HER2 ECD, distinct from the binding site of trastuzumab on domain IV. Consequently, it potently inhibits HER2 as well as the dimerisation of HER2 with other HER family receptors, including HER3 [6]. The growing body of preclinical evidence supporting the central role of the HER2-HER3 interaction in driving PI3K/Akt-mediated tumorigenesis in HER2-overexpressing breast cancers strengthens the scientific rationale behind the development of this class of agents [26].

 Pertuzumab combined with trastuzumab results in concurrent blockade of multiple HER family members, and their synergistic activity has been demonstrated in HER2-overexpressing breast cancer xenograft models [44]. Dual inhibition with pertuzumab and trastuzumab has reached an advanced phase of development in combination with chemotherapy. A randomised phase III study (CLEOPATRA, *NCT00567190*) evaluated the benefit of adding pertuzumab to the combination of trastuzumab and docetaxel in previously untreated HER2-positive metastatic breast cancer. Although no comprehensive data has been released, preliminary results of the recently completed study indicate that the primary endpoint of PFS was significantly prolonged in the experimental pertuzumab-containing arm. These potentially practice-changing findings have led Genentech/Hoffman-La Roche to seek regulatory approval for the drug combination [45]. A similarly designed randomised phase III study evaluating the addition of pertuzumab to trastuzumab plus chemotherapy will soon commence in the adjuvant setting (*NCT01358877*).

 The dual anti-HER2 combination of pertuzumab and trastuzumab has been evaluated without chemotherapy in the open-label, phase II BO17929 study conducted on HER2-positive metastatic breast cancer patients with prior trastuzumab failure, yielding extremely promising results. Complete response rates, ORR, and CBR were 7.6%, 24.2%, and 50%, respectively, and the median PFS was 5.5 months. The combination was well tolerated, and importantly, the incidence of cardiac dysfunction was minimal [46]. The rationale for developing pertuzumab in combination with trastuzumab was reinforced by data from a separate arm of the same study, which showed that 14.3% of patients responded to the reintroduction of trastuzumab upon disease progression on pertuzumab monotherapy, demonstrating that the two HER2-directed agents have synergistic, nonoverlapping mechanisms of actions [47]. Further support for the potential development of this chemotherapy-free anti-HER2 combination was derived from the recently completed phase II NeoSphere study, which evaluated the efficacy of preoperative pertuzumab, trastuzumab, or their combination, with or without docetaxel in HER2-positive early-stage breast cancer. Similar to what has been reported in neoALTTO, pCR rates were highest with docetaxel plus dual anti-HER2 blockade compared to docetaxel with either trastuzumab or pertuzumab alone (45.8% *versus* 29% *versus *24%, *P* < 0.014). Interestingly, a promising pCR rate of 17.8% was achieved with dual anti-HER2 blockade in the absence of chemotherapy, further highlighting the potential of combining two anti-HER2 agents with different mechanisms of action [48].

#### 3.2.2. Antibody-Drug Conjugate (ADC), Trastuzumab-DM1

T-DM1 is the first and only HER2-directed ADC in clinical development and combines the intracellular delivery of a microtubule-depolymerisation agent, DM1, a maytansine derivative, with the antitumour activity of trastuzumab. Upon binding to the HER2 receptor, T-DM1 is internalised and DM1 released intracellularly, enabling the selective delivery of the potent cytotoxic agent to HER2-overexpressing cells with limited systemic toxicity [6, 49]. Preclinical studies have demonstrated the enhanced efficacy of this agent compared to unconjugated trastuzumab in both trastuzumab-sensitive as well as in trastuzumab-resistant HER2-overexpressing tumour models [50, 51].

Excellent response rates of 40% to T-DM1 have been reported in an open-label, single-arm, phase II study conducted in patients with HER2-positive metastatic breast cancer, all of whom had progressed on trastuzumab-based therapy and 40% of whom had received prior lapatinib therapy. Severe toxicities including grade 4 thrombocytopenia and transaminitis were rare at 6% [52]. The high proportion of patients achieving objective responses in this study has allayed concerns that further antibody-based treatment may be rendered ineffective due to the proteolytic cleavage of HER2 following prior trastuzumab exposure [51].

Multiple clinical trials have been designed to compare the efficacy of single-agent T-DM1 with existing HER2-directed therapies combined with chemotherapy in patients with HER2-positive metastatic breast cancer. Preliminary results of a randomised phase II study (TDM4450g) of T-DM1 versus trastuzumab plus docetaxel (TH) in the first-line treatment of HER2-positive metastatic breast cancer patients were recently released. While response rates were comparable in the two arms (48% *versus* 41%; T-DM1 *versus* TH, resp.), T-DM1 was much less toxic, with grade 3 and above toxicities approximately half that of TH (37.3% *versus* 75%) [53]. The EMILIA study, a multicentre, open-label, randomised, phase III study comparing the efficacy of T-DM1 versus the combination of capecitabine and lapatinib in patients who have received prior taxane- and trastuzumab-based regimens, is nearing completion (*NCT00829166*). In addition, the role of T-DM1 delivered sequentially with anthracycline-based chemotherapy is being explored in the adjuvant/neoadjuvant setting in patients with HER2-positive early breast cancer (*NCT01196052*).

 Based on preclinical research demonstrating the synergistic antitumour activity of T-DM1 and pertuzumab in a trastuzumab-resistant breast cancer xenograft model [54], a phase Ib/II study of the combination has been performed, demonstrating tolerability and encouraging efficacy, with an ORR of 35.7% in a heavily pretreated HER2-positive metastatic breast cancer population [55]. Further to this, a three-arm randomised phase III study (MARIANNE, *NCT01120184*) is underway to evaluate T-DM1 alone and T-DM1 combined with pertuzumab versus the reference arm of trastuzumab plus taxane in previously untreated patients with HER2-positive metastatic breast cancer.

#### 3.2.3. Tyrosine Kinase Inhibitors

Since trastuzumab resistance may arise from cross-talk among other HER proteins resulting in lateral activation and incomplete inhibition of downstream signalling, one approach to overcoming trastuzumab resistance is the simultaneous inhibition of multiple HER receptors [56]. Several novel HER2 small-molecule TKIs have demonstrated promising activity and are in various stages of clinical development.

**Neratinib (HKI-272)**

Neratinib, a potent, low-molecular-weight, orally administered, irreversible pan-HER (HER1, HER2 and HER4) receptor TKI, is one of the most advanced novel HER TKI in clinical development and offers more complete HER receptor blockade than lapatinib which inhibits only HER1 and HER2. An early preclinical study demonstrated that the compound inhibited cell proliferation of HER2-overexpressing breast cancer cell lines and xenografts through downregulation of the MAP kinase and PI3K/Akt pathways and induced cell cycle arrest [57].

Clinical studies have evaluated neratinib as a single agent or in combination with trastuzumab or chemotherapy. A recently published phase II, open-label study evaluated the efficacy and tolerability of neratinib monotherapy in two cohorts of patients with HER2-positive metastatic breast cancer: those with and those without prior trastuzumab treatment. The efficacy results of the two cohorts were a 16-week PFS (the primary endpoint) of 59% and 78%, respectively, and an ORR of 24% and 56%, respectively, while diarrhoea was a common but manageable toxicity [58]. Despite the caveats of cross-trial comparisons, these results seem much more promising than those of a phase II study of lapatinib monotherapy in HER2-positive metastatic breast cancer patients with prior trastuzumab treatment, where ORR was only 4.3%, and median PFS was 9 weeks [59], signifying that neratinib has a potential role as a single agent, as opposed to lapatinib, which is seldom administered as monotherapy. Several studies are ongoing to address pertinent issues regarding the therapeutic role of neratinib, including its efficacy in metastatic disease as single agent in a randomised phase II open-label study of neratinib versus the combination of lapatinib and capecitabine in patients with prior trastuzumab and taxane exposure (*NCT00777101*) and in combination with chemotherapy in a randomised phase II study (NEFERTT, *NCT00915018*) comparing neratinib and paclitaxel versus trastuzumab and paclitaxel in previously untreated metastatic disease. It is also being evaluated in early-stage disease both in the neoadjuvant and adjuvant settings (*NCT01008150 and NCT00878709*).

Preliminary data from a phase I/II study of the combination of neratinib and trastuzumab in HER2-positive metastatic breast cancer patients with prior trastuzumab exposure demonstrated both tolerability and efficacy, with an ORR of 27%, 16-week PFS of 47%, and median PFS of 19 weeks [60]. These data warrant further investigations to clarify the utility of this anti-HER2 combination.

**Other HER2-Targeted TKIs**

While afatinib (BIBW-2992) targets the same receptors as lapatinib, it does so in an irreversible manner, resulting in more sustained inhibition of EGFR and HER2. It has demonstrated activity in trastuzumab-resistant HER2-overexpressing cell lines as well as promising clinical activity in phase I development [61]. Its potential was confirmed in a phase II study of single-agent afatinib, which yielded 12% response rate in a heavily pre-treated population of HER2-positive metastatic breast cancer patients with prior trastuzumab exposure [62]. An ongoing phase III randomised study (LUX-Breast 1) is assessing the efficacy of afatinib plus vinorelbine versus the continuation of trastuzumab plus vinorelbine in HER2-positive metastatic breast cancer following trastuzumab failure (*NCT01125566*).

Two other orally active TKIs, ARRY-380 and ARRY-334543, are in the process of development by Array BioPharma. ARRY-380 is a reversible and selective HER2 TKI which has demonstrated significant dose-related tumour growth inhibition superior to that of trastuzumab and lapatinib in preclinical studies, as well as promising efficacy in an ongoing phase I expansion study in HER2-overexpressing solid tumours, including activity in p95HER2-overexpressing tumours (*NCT00650572*) [63]. ARRY-334543, a reversible pan-HER TKI which has demonstrated significant preclinical activity in HER2-overexpressing tumour models and synergism with trastuzumab and docetaxel [64], is currently being evaluated in early phase studies in advanced solid tumours (*NCT00710736*).

#### 3.2.4. Heat-Shock Protein 90 Inhibitors

HSP90 belongs to a family of chaperone proteins which facilitate the conformational maturation and folding of various signalling proteins, including HER2. In a preclinical study, interference with its function led to ubiquitylation and proteasomal degradation of HER2 and the resultant abrogation of the PI3K/Akt pathway, in turn causing growth inhibition of HER2-overexpressing tumours in murine xenograft models [65]. A potential therapeutic niche for HSP90 inhibitors in the setting of trastuzumab resistance has been described, where administration of HSP90 inhibitors resulted in down-regulation of truncated p95HER2 and inhibition of cell proliferation in p95HER2-overexpressing trastuzumab-resistant breast tumour models [66].

 At least three HSP90 inhibitors have undergone clinical investigation in HER2-positive breast cancer, and to date, all have been evaluated in combination with trastuzumab. One promising compound is tanespimycin (17-AAG/KOS-953), which has demonstrated significant activity and tolerability in combination with trastuzumab in a phase II study conducted in advanced trastuzumab-refractory HER2-positive breast cancer, with an ORR of 24%, CBR of 59%, median PFS of 6 months, and median OS of 17 months [67]. These data undoubtedly warrant further exploration of its therapeutic role in phase III studies. Ironically, a structurally related biologically active form of 17-AAG, retaspimycin (IPI-504), did not share the same success despite promising preclinical activity in trastuzumab-resistant HER2-overexpressing cell lines; while treatment in combination with trastuzumab in a phase II study resulted in modest clinical activity in heavily pretreated patients previously exposed to trastuzumab, it did not meet the prespecified efficacy criteria for trial expansion [68]. Another HSP90 inhibitor, AUY922, is currently being evaluated in combination with trastuzumab in patients with trastuzumab-refractory HER2-positive metastatic breast cancer in a phase I/II study (*NCT01271920*). 

#### 3.2.5. Mammalian Target of Rapamycin (mTOR)/PI3K Inhibitors

PTEN loss leads to the constitutive activation of Akt, which in turn activates mTOR in the PI3K/Akt signalling pathway. Two preclinical studies suggest the potential of this pathway as a therapeutic target; HER2-overexpressing breast cancer cell lines and xenograft models transfected with loss-of-function PTEN mutations and activating PIK3CA mutations resulted in trastuzumab and lapatinib resistance which was effectively reversed by the PI3K/mTOR inhibitor, NVP-BEZ235 [21, 69].

**Everolimus**

Everolimus is the most advanced mTOR inhibitor undergoing clinical investigation and is being developed in combination with existing HER2-directed therapies with or without chemotherapy. A recently published phase I/II study evaluated the chemotherapy-free combination of everolimus and trastuzumab in heavily pretreated HER2-positive metastatic breast cancer patients with prior trastuzumab exposure, which yielded an ORR of 15%, CBR of 34%, and median PFS of 4.1 months. The overall safety profile was acceptable, but did include a less than 10% incidence of grade 3 diarrhoea, fatigue, and stomatitis [70]. The combination of everolimus and lapatinib is currently being investigated in a phase II study of HER2-positive metastatic breast cancer patients with prior trastuzumab exposure (*NCT01283789*).

The combination of everolimus with trastuzumab and weekly paclitaxel appeared highly active in heavily pre-treated HER2-positive metastatic breast cancer patients with prior trastuzumab and taxane exposure, yielding an ORR of 25%, stable disease (SD) rate of 56%, and acceptable toxicity profile in a multicentre phase II study [71]. Two placebo-controlled randomised phase III studies are in progress to evaluate the benefit of adding everolimus to trastuzumab and paclitaxel in the first-line metastatic setting (BOLERO-1) and to trastuzumab and vinorelbine in patients with prior trastuzumab and taxane exposure (BOLERO-3) (*NCT00876395, NCT01007942*).

**Other mTOR/PI3K Pathway Inhibitors**

Apart from everolimus, two other rapamycin analogues in the developmental pipeline in the treatment of HER2-positive breast cancers are deforolimus (AP23573) and temsirolimus (CCI-779). A phase II study of oral deforolimus in combination with trastuzumab has recently been completed in HER2-overexpressing metastatic breast cancer patients with prior trastuzumab exposure (NCT00736970). Compensatory increase in HER3 signalling with inhibition of the PI3K/Akt signalling pathway supports the novel approach of dual inhibition with an mTOR inhibitor and a pan-HER receptor TKI [26], and the combination of temsirolimus and neratinib has demonstrated tolerability and highly promising activity in a phase I study [72] and is currently being evaluated in an ongoing phase II study (*NCT01111825*).

Novartis is currently conducting early-phase studies of two PI3K inhibitors that compete reversibly with the ATP-binding site of the PI3K p110 catalytic subunit. BKM120, a pan-class I PI3K inhibitor, is being assessed in combination with trastuzumab in a phase Ib/II study of HER2-overexpressing breast cancer patients with prior trastuzumab exposure (*NCT01132664*), while BEZ235, a dual PI3K and mTOR inhibitor, is planned for evaluation as a single-agent in a phase II study (*NCT01288092*) [73, 74].

#### 3.2.6. Angiogenesis Inhibitors

**Monoclonal Antibodies against VEGF**

The rationale for the simultaneous blockade of both HER2 and VEGF pathways is based on preclinical data demonstrating upregulation of angiogenesis in HER2-overexpressing breast tumours, and clinical data demonstrating that overexpression of both HER2 and VEGF was associated with a poorer prognosis in breast cancer patients in a large retrospective analysis [75]. Following the results of a phase II study which demonstrated encouraging efficacy (ORR 46%) and tolerability of bevacizumab and trastuzumab in the first-line treatment of HER2-positive metastatic breast cancer [76], the role of adding bevacizumab, a monoclonal antibody against VEGF, to the combination of trastuzumab and taxane-based chemotherapy is being assessed in two large randomised phase III studies, AVEREL (*NCT00391092*) and ECOG1105 (*NCT00520975*). The same strategy is also being evaluated in the adjuvant setting in the ongoing BETH study (*NCT00625898*) [77].

**VEGF-Receptor (VEGFR) TKIs**

A phase II open-label study assessed the safety and efficacy of adding pazopanib, an oral angiogenesis inhibitor targeting VEGFR, platelet-derived growth factor receptor and c-kit, to lapatinib in previously untreated HER2-positive metastatic breast cancer. Results showed lower 12-week progressive disease (19% versus 27%) and better response rates (44% versus 30%) with the combination compared to lapatinib alone, and whilst toxicities of diarrhoea and abnormal liver function tests were greater in the combination arm, they were still regarded as tolerable [78]. This has led to an ongoing randomised phase III study comparing the combination of pazopanib and lapatinib versus lapatinib monotherapy in patients with HER2-overexpressing inflammatory breast cancer (*NCT00558103*).

#### 3.2.7. IGF-1R Inhibitors

Bidirectional cross-talk between the IGF-1R and HER2 signalling pathways is one mechanism of trastuzumab resistance, and the human anti-IGF-1R antibody, CP-751871 (*Pfizer*) and small-molecule selective IGF-1R TKI, NVP-AEW541 (*Novartis*) have demonstrated antitumour activity against trastuzumab-resistant breast cancer tumour models [79, 80]. Two IGF-1R inhibitors are being evaluated in combination with existing HER2-directed therapies for the treatment of HER2-positive metastatic breast cancer in clinical studies. Cixutumumab (IMC-A12), an IGF-1R monoclonal antibody, is being assessed in combination with lapatinib and capecitabine in patients with prior trastuzumab, anthracycline, and/or taxane exposure in a randomised phase II study (*NCT0068493*), whilst the combination of trastuzumab and BMS-754807, a small-molecule, reversible IGF-1R TKI, is undergoing evaluation in patients with prior trastuzumab failure in a phase I/II study (*NCT00788333*).

#### 3.2.8. Bispecific Antibodies

Continuing strategies to overcome trastuzumab resistance include the development of a class of agents known as trifunctional, bispecific antibodies. MM-111 is an antibody targeting both the HER2/HER3 heterodimer and the HER3 ligand, heregulin, and is currently being evaluated in combination with trastuzumab in HER2-overexpressing metastatic breast cancer patients with prior trastuzumab exposure (*NCT01097460*) [81]. A phase II study of another bispecific antibody against HER2 and CD3, ertumaxomab, in trastuzumab-refractory HER2-positive metastatic breast cancer was however terminated after the company halted the development of the compound due to strategic changes (*NCT00452140*). 

#### 3.2.9. Novel Chemotherapy-Free Anti-HER2 Combinations

Rational combinations of novel anti-HER2 therapies are of particular interest not only because of their ability to overcome trastuzumab resistance through their concurrent blockade of multiple HER2 family members, but also for their potential to offer chemotherapy-free therapeutic options that are relatively less toxic. The combination of trastuzumab and lapatinib has recently been integrated into clinical practice [42]. Most other combinations in development include combining a novel anti-HER2 agent with trastuzumab. Of these, the most convincing evidence for the benefit of dual inhibition over single-agent therapy is derived from the randomised phase II studies, BO17929 (*NCT00301899*) and NeoSphere [48], which strongly support the synergistic activity of pertuzumab and trastuzumab. Neratinib combined with trastuzumab has similarly demonstrated promising efficacy [60]. All the HSP inhibitors and most of the mTOR inhibitors under evaluation in HER2-positive breast cancer have been developed in combination with trastuzumab, with studies supporting their efficacy in the absence of chemotherapy, although it would also be of interest to study these agents as monotherapy to define if trastuzumab is required for maximal efficacy [6]. Lastly, the combination of an mTOR inhibitor and novel HER-receptor TKI is worthy of mention due to its strong preclinical rationale and promising early-phase activity [72]. Apart from dual inhibition using two HER2-directed therapies, synergism has been demonstrated between anti-HER2 agents and angiogenesis inhibitors, with lapatinib and pazopanib being a promising chemotherapy-free combination. The development of even more robust chemotherapy-free anti-HER2 combinations, driven by a clearer understanding of basic scientific mechanisms, is highly anticipated to improve HER2-directed therapeutic strategies in the future.

### 3.3. Future Challenges and Directions

With the emergence of several novel HER2-directed agents, recent therapeutic advancements have been remarkable compared to the limited progress following the initial regulatory approval of trastuzumab more than a decade ago. Improved insights into the mechanisms of trastuzumab resistance have led to new treatment strategies employing dual anti-HER2 blockade, which have improved response rates and progression-free survival.

While the substantial amount of research dedicated to the development of novel ant-HER2 agents is laudable, the vast array of treatment options in an ever-changing landscape of anti-HER2 therapy may raise more questions than answers. Clinicians will be continually challenged to design optimal combinations and sequences of anti-HER2 agents, chemotherapeutic agents, and even endocrine therapy for the individualised treatment of patients with HER2-positive breast cancer. These issues can rarely be fully addressed through the simplistic design of current therapeutic trials. At present, a physician treating an individual who has progressed on first-line trastuzumab-based therapy already faces the dilemma of whether the patient should receive further trastuzumab together with an alternative chemotherapeutic agent, continue trastuzumab in combination with lapatinib, or switch to lapatinib. The growing number of HER2-directed therapies in the pipeline will only add to the complexity of this decision-making process. In addition, a large proportion of HER2-positive patients relapsing with metastatic disease in the future will have received adjuvant trastuzumab or even lapatinib therapy, indicating that new treatment paradigms will be required for this group of patients.

The extensive preclinical efforts in elucidating mechanisms of trastuzumab resistance should translate into individualised therapy through biomarker-driven approaches. However, this would first require the development of validated assays for the accurate measurement of resistance factors in archival tissue specimens, and to date, advancements in this arena have been slow [24]. For example, despite the fact that HER2 truncation is the best-studied mechanism of trastuzumab resistance, monoclonal antibodies to analyse p95HER2 levels in tumour samples have only recently become available [4, 82]. The design of future prospective clinical trials in HER2-positive breast cancer should ideally be guided by predictive biomarkers. However, this significantly increases the complexity of randomised studies since a variety of mechanisms of resistance may exist in a patient population, and in reality, it is likely to take years before trastuzumab resistance biomarker-directed treatment approaches may be integrated into routine clinical practice.

### 3.4. Conclusion

Trastuzumab resistance poses a significant challenge in the treatment of HER2-positive breast cancer. Substantial research has been dedicated to elucidating mechanisms of trastuzumab resistance, as well as developing a myriad of novel anti-HER2 therapeutic agents with promising clinical activity. This has resulted in a major paradigm shift in the treatment of HER2-positive breast cancer and has given rise to a rapidly expanding range of therapeutic options in active clinical development. Future efforts should be directed towards biomarker-driven, HER2-directed therapies for optimal selection of therapy for the individual patient.

## Figures and Tables

**Table 1:** Pertinent clinical trials of novel HER2-directed therapies in HER2-positive breast cancer.

Trial	Development phase (^a^ *Protocol No.*)	Study population (sample size/planned enrollment)	Agents under evaluation
	**Dimerisation inhibitor (Pertuzumab)**/*Genentech *		

**CLEOPATRA**	**Randomised phase III *(NCT00567190) ***	^ b^ **MBC, 1st line **(*n* = 808)	**Docetaxel + trastuzumab + pertuzumab *versus *docetaxel + trastuzumab + placebo**
BO17929 [46]	Phase II	MBC, ≥2nd line, prior trastuzumab (*n* = 85)	Pertuzumab + trastuzumab pertuzumab → pertuzumab + trastuzumab on ^c^PD
	**Randomised phase III *(NCT01358877) ***	**Adjuvant, ** ^ d^ **EBC **(*n* = 3806)	**Chemotherapy + trastuzumab + pertuzumab *versus* chemotherapy + trastuzumab + placebo**
NeoSphere [48]	Randomised phase II	Neoadjuvant, stage II/III EBC (*n* = 417)	Docetaxel + trastuzumab + pertuzumab *versus* docetaxel + pertuzumab *versus* docetaxel + trastuzumab *versus* pertuzumab + trastuzumab

	**Antibody-Drug Conjugate (Trastuzumab-DM1)**/*Genentech *		

**MARIANNE**	**Randomised phase III *(NCT01120184) ***	**MBC, 1st line **(*n* = 1092)	**T-DM1 + pertuzumab *versus* T-DM1 + placebo *versus* trastuzumab + taxane**
**EMILIA**	**Randomised phase III *(NCT00829166) ***	**MBC, ≥2nd line, prior trastuzumab + taxane **(*n* = 980)	**T-DM1 *versus* lapatinib + capecitabine**
TDM4450g [53]	Randomised phase II (*NCT00679341) *	MBC, 1st line (*n* = 137)	T-DM1 *versus* trastuzumab + docetaxel
	**Single-arm phase II *(NCT01196052) ***	**Neoadjuvant/adjuvant, stage I-III **(*n* = 135) **after anthracyclines**	**T-DM1 **

	**Novel Tyrosine Kinase Inhibitors **		

	(i) Neratinib (HK-272)/*Pfizer *		

**NEFERTT **	**Randomised phase II *(NCT00915018) ***	**MBC, 1st line **(*n* = 480)	**Neratinib + paclitaxel *versus* trastuzumab + paclitaxel**
	**Randomised phase II *(NCT00777101) ***	**MBC, ≥2nd line, prior trastuzumab + taxane **(*n* = 233)	**Neratinib monotherapy *versus* lapatinib + capecitabine**
Reference [60]	Single-arm phase II *(NCT00398567) *	MBC, ≥2nd line, prior trastuzumab (*n* = 45)	Neratinib + trastuzumab
**ExteNET**	**Randomised phase III *(NCT00878709) ***	**Adjuvant, node-positive, stage II-III **(*n* = 3850) **Completed trastuzumab**	**Neratinib monotherapy *versus* placebo**
**FB-7**	**Randomised phase II *(NCT01008150) ***	**Neoadjuvant, stage IIB-IIIC **(*n* = 120)	**Neratinib + paclitaxel *versus* trastuzumab + paclitaxel**

	(ii) Afatinib (BIBW-2992)/*Boehringer Ingelheim *		

**LUX-Breast 1**	**Randomised phase III *(NCT01125566) ***	**MBC, ≥2nd line, prior trastuzumab **(*n* = 780)	**Afatinib + vinorelbine *versus* trastuzumab + vinorelbine**
Reference [62]	Single-arm Phase II *(NCT00431067) *	MBC, ≥2nd line, prior trastuzumab (*n* = 41)	Afatinib monotherapy

	(iii) ARRY-380/*Array BioPharma *		

	**Expansion phase I *(NCT00650572) ***	**MBC, ≥2nd line **(*n* = 50)	**ARRY-380 monotherapy**

	(iv) ARRY-334543/*Array BioPharma *		

	**Phase I *(NCT00710736) ***	**Advanced solid tumours **(*n* = 29)	**ARRY-334543 + capecitabine**

	**Heat-shock protein 90 inhibitors**		

	(i) Tanespimycin (17-AAG)/*Bristol-Myers Squibb *		

Reference [67]	Single-arm phase II *(NCT00773344) *	MBC, ≥2nd line, prior trastuzumab (*n* = 29)	Tanespimycin + trastuzumab

	(ii) AUY922/*Novartis *		

	**Phase Ib/II *(NCT001271920) ***	**MBC, ≥2nd line, prior trastuzumab **(*n* = 45)	**AUY 922 + trastuzumab**

	**mTOR inhibitors**		

	(i) Everolimus (RAD001)/*Novartis *		

**BOLERO-1 **	**Randomised phase III *(NCT00876395) ***	**MBC, 1st line **(*n* = 717)	**Paclitaxel + trastuzumab + everolimus *versus* paclitaxel + trastuzumab + placebo **
**BOLERO-3 **	**Randomised phase III *(NCT01007942) ***	**MBC, ≥2nd line, prior trastuzumab + taxane **(*n* = 572)	**Vinorelbine + trastuzumab + everolimus *versus* vinorelbine + trastuzumab + placebo**
Reference [70]	Phase I/II *(NCT00426566) *	MBC, ≥2nd line, prior trastuzumab (*n* = 55)	Everolimus + trastuzumab
	**Single-arm phase II *(NCT01283789) ***	**MBC, ≥2nd line, prior trastuzumab **(*n* = 45)	**Everolimus + lapatinib**

	(ii) Deforolimus (AP23573)/*Ariad *		

	Single-arm Phase II *(NCT00736970) *	MBC, ≥2nd line, prior trastuzumab (*n* = 34)	Deforolimus + trastuzumab

	(iii) Temsirolimus (CCI-779)/*Wyeth *		

	**Single-arm phase I/II *(NCT01111825) ***	**MBC, ≥2nd line, prior trastuzumab **(*n* = 65)	**Temsirolimus + neratinib**

	**PI3K inhibitors **		

	(i) BKM120/*Novartis *		

	**Single-arm phase Ib/II *(NCT01132664) ***	**MBC, ≥2nd line, prior trastuzumab **(*n* = 70)	**BKM120 + trastuzumab **

	(ii) BEZ235/*Novartis *		

	**Single-arm phase II *(NCT01288092) ***	**MBC, ≥2nd line, prior trastuzumab **(*n* = 120)	**BEZ235 monotherapy **

	**Angiogenesis inhibitors**		

	(i) Bevacizumab/*Genentech/Roche *		

**AVEREL**	**Randomised phase III *(NCT00391092) ***	**MBC, 1st line **(*n* = 407)	**Docetaxel + trastuzumab + bevacizumab *versus* docetaxel + trastuzumab**
**ECOG1105**	**Randomised phase III *(NCT00520975) ***	**MBC, 1st line **(*n* = 489)	**Carboplatin + paclitaxel + trastuzumab** **+ bevacizumab *versus* carboplatin + paclitaxel + trastuzumab + placebo**
**BETH**	**Randomised phase III *(NCT00625898) ***	**Adjuvant, EBC **(*n* = 3509)	**Chemotherapy + trastuzumab + bevacizumab *versus* chemotherapy + trastuzumab**

	(ii) Pazopanib*/GlaxoSmithKline *		

Reference [78]	Randomised phase II *(NCT00347919) *	MBC, 1st line (*n* = 62)	Pazopanib + lapatinib *versus* lapatinib
	**Randomised phase III *(NCT00558103) ***	**Inflammatory breast cancer, ≥2nd line **(*n* = 360) **Prior trastuzumab/chemotherapy**	**Pazopanib + lapatinib *versus* lapatinib **

	**IGF-1R inhibitors**		

	(i) Cixutumumab (IMC-A12)/*Eli Lilly *		

	**Randomised phase II *(NCT00684983) ***	**MBC, ≥2nd line, prior trastuzumab, anthracycline and/or taxane **(*n* = 154)	**Lapatinib + capecitabine + cixutumumab *versus *** ** lapatinib + capecitabine**

	(ii) BMS-754807*/Bristol-Myers Squibb *		

	**Phase I/II *(NCT00788333) ***	**MBC, ≥2nd line, prior trastuzumab **(*n* = 48)	**BMS-754807 + trastuzumab**

	**Bispecific antibody (MM-111)**/*Merrimack Pharmaceuticals *		

	**Phase I/II *(NCT01097460) ***	**MBC, ≥2nd line, prior trastuzumab **(*n* = 50)	**MM-111 + trastuzumab**

*Bold rows denote ongoing studies or trials pending results, ^a^ *ClinicalTrials.gov*-http://clinicaltrials.gov/,^b^ *metastatic breast cancer*, ^c^ *disease progression*, ^d^ *early breast cancer*.
